# Supplementary material for: MetaRibo-Seq measures translation in microbiomes
Source: Nat Commun. 2020 Jun 29;11:3268. doi: 10.1038/s41467-020-17081-z (PMC7324362; doi:10.1038/s41467-020-17081-z)
Supplement: Supplementary file 10 — Supplementary Data 7 [file 41467_2020_17081_MOESM10_ESM.zip › File2/Confidence_High_Taxonomy/319809_out.krona.html]

Javascript must be enabled to view this page.

members
magnitude
magnitudeUnassigned
count
unassigned
taxon
rank

319809\_out

331

superkingdom
2
331

331
phylum
1239

331
class
186801

331
186802
order

331
541000
family

genus
216851
331


SRS011134\_contig\_number\_38837SRS011239\_contig\_number\_18798SRS011302\_contig\_number\_317SRS012849\_contig\_number\_30553SRS013098\_contig\_number\_41170SRS013158\_contig\_number\_5497SRS013476\_contig\_number\_30532SRS013521\_contig\_number\_5102SRS013638\_contig\_number\_19686SRS013687\_contig\_number\_5107SRS013940\_contig\_number\_22002SRS013951\_contig\_number\_377SRS013965\_contig\_number\_2703SRS013965\_contig\_number\_40453SRS014235\_contig\_number\_45154SRS014287\_contig\_number\_954SRS014313\_contig\_number\_5760SRS014412\_contig\_number\_14862SRS014415\_contig\_number\_contig-100\_1065.1066SRS014459\_contig\_number\_19897SRS014613\_contig\_number\_19384SRS014683\_contig\_number\_contig-100\_1116.120744SRS014855\_contig\_number\_6000SRS014923\_contig\_number\_46926SRS014948\_contig\_number\_20291SRS014979\_contig\_number\_28728SRS015065\_contig\_number\_17557SRS015264\_contig\_number\_2658SRS015578\_contig\_number\_176SRS015579\_contig\_number\_25119SRS015663\_contig\_number\_17321SRS015694\_contig\_number\_25169SRS015782\_contig\_number\_44164SRS015794\_contig\_number\_9689SRS015854\_contig\_number\_24057SRS015890\_contig\_number\_contig-100\_943.164113SRS015960\_contig\_number\_22031SRS016018\_contig\_number\_14284SRS016056\_contig\_number\_6382SRS016203\_contig\_number\_13401SRS016335\_contig\_number\_49901SRS016495\_contig\_number\_17869SRS016753\_contig\_number\_3465SRS016840\_contig\_number\_2563SRS016954\_contig\_number\_22986SRS016989\_contig\_number\_contig-100\_997.102341SRS017103\_contig\_number\_29018SRS017191\_contig\_number\_20702SRS017247\_contig\_number\_5376SRS017307\_contig\_number\_23803SRS017433\_contig\_number\_17484SRS017521\_contig\_number\_15657SRS017701\_contig\_number\_contig-100\_1939.120452SRS017821\_contig\_number\_25180SRS017859\_contig\_number\_contig-100\_657.64894SRS018313\_contig\_number\_9034SRS018351\_contig\_number\_10769SRS018427\_contig\_number\_contig-100\_1298.187462SRS018575\_contig\_number\_1959SRS018623\_contig\_number\_19021SRS018656\_contig\_number\_contig-100\_989.80081SRS018817\_contig\_number\_24747SRS018836\_contig\_number\_26833SRS018888\_contig\_number\_3375SRS018936\_contig\_number\_11442SRS018984\_contig\_number\_34063SRS019030\_contig\_number\_19458SRS019161\_contig\_number\_42318SRS019285\_contig\_number\_contig-100\_1087.1088SRS019286\_contig\_number\_contig-100\_1053.50186SRS019381\_contig\_number\_contig-100\_1045.135968SRS019397\_contig\_number\_9994SRS019445\_contig\_number\_31874SRS019496\_contig\_number\_11579SRS019601\_contig\_number\_contig-100\_1730.153283SRS019685\_contig\_number\_21395SRS019693\_contig\_number\_3774SRS019787\_contig\_number\_2017SRS019808\_contig\_number\_18106SRS019910\_contig\_number\_16909SRS019968\_contig\_number\_18248SRS020233\_contig\_number\_52794SRS020328\_contig\_number\_38196SRS020869\_contig\_number\_39261SRS021219\_contig\_number\_contig-100\_4139.71997SRS021484\_contig\_number\_2831SRS021948\_contig\_number\_32750SRS022071\_contig\_number\_26809SRS022137\_contig\_number\_18883SRS022524\_contig\_number\_contig-100\_932.86951SRS022609\_contig\_number\_46774SRS023583\_contig\_number\_5364SRS023715\_contig\_number\_contig-100\_1531.170760SRS023914\_contig\_number\_29305SRS023971\_contig\_number\_1808SRS024009\_contig\_number\_10134SRS024075\_contig\_number\_15566SRS024132\_contig\_number\_40604SRS024331\_contig\_number\_16368SRS024388\_contig\_number\_contig-100\_766.35432SRS024435\_contig\_number\_45414SRS024492\_contig\_number\_14102SRS024625\_contig\_number\_20358SRS024663\_contig\_number\_15894SRS042284\_contig\_number\_3172SRS042628\_contig\_number\_51SRS042966\_contig\_number\_19270SRS043001\_contig\_number\_10687SRS043411\_contig\_number\_12000SRS043701\_contig\_number\_contig-100\_663.118235SRS043768\_contig\_number\_32928SRS043841\_contig\_number\_11661SRS044535\_contig\_number\_24724SRS045004\_contig\_number\_contig-100\_1616.182723SRS045195\_contig\_number\_6148SRS045244\_contig\_number\_9024SRS045645\_contig\_number\_31423SRS045739\_contig\_number\_11307SRS045826\_contig\_number\_23435SRS046068\_contig\_number\_5150SRS046369\_contig\_number\_11843SRS046502\_contig\_number\_6889SRS046712\_contig\_number\_2692SRS046717\_contig\_number\_10713SRS047014\_contig\_number\_25215SRS047044\_contig\_number\_19327SRS047433\_contig\_number\_25253SRS048060\_contig\_number\_3254SRS048164\_contig\_number\_13786SRS048870\_contig\_number\_22446SRS049402\_contig\_number\_12103SRS049446\_contig\_number\_13680SRS049712\_contig\_number\_11026SRS049773\_contig\_number\_28542SRS049900\_contig\_number\_20291SRS049995\_contig\_number\_37413SRS050026\_contig\_number\_contig-100\_7647.43986SRS050422\_contig\_number\_30168SRS050752\_contig\_number\_20355SRS050801\_contig\_number\_3440SRS050925\_contig\_number\_31332SRS050941\_contig\_number\_12667SRS050998\_contig\_number\_16739SRS051031\_contig\_number\_28065SRS051610\_contig\_number\_8662SRS051882\_contig\_number\_4513SRS052027\_contig\_number\_20631SRS052078\_contig\_number\_4843SRS052697\_contig\_number\_8085SRS053214\_contig\_number\_21037SRS053335\_contig\_number\_14696SRS053356\_contig\_number\_41098SRS053398\_contig\_number\_22482SRS053573\_contig\_number\_2539SRS053649\_contig\_number\_contig-100\_2679.129348SRS054905\_contig\_number\_25240SRS054956\_contig\_number\_1050SRS055017\_contig\_number\_29678SRS055982\_contig\_number\_14414SRS056259\_contig\_number\_29941SRS056273\_contig\_number\_30868SRS056519\_contig\_number\_7595SRS057480\_contig\_number\_contig-100\_270.80930SRS058070\_contig\_number\_32169SRS058145\_contig\_number\_contig-100\_2483.37086SRS058723\_contig\_number\_7747SRS058770\_contig\_number\_2103SRS062427\_contig\_number\_10307SRS062654\_contig\_number\_10103SRS062701\_contig\_number\_1549SRS063040\_contig\_number\_20713SRS063190\_contig\_number\_15316SRS063370\_contig\_number\_13789SRS063518\_contig\_number\_36600SRS063813\_contig\_number\_6433SRS063985\_contig\_number\_22057SRS064276\_contig\_number\_16642SRS064757\_contig\_number\_24350SRS064973\_contig\_number\_1233SRS065397\_contig\_number\_13084SRS065504\_contig\_number\_40384SRS074670\_contig\_number\_8949SRS074964\_contig\_number\_5031SRS075341\_contig\_number\_24351SRS075716\_contig\_number\_4865SRS075773\_contig\_number\_30213SRS075821\_contig\_number\_15080SRS075878\_contig\_number\_24043SRS075984\_contig\_number\_20533SRS076756\_contig\_number\_19961SRS076804\_contig\_number\_18679SRS077024\_contig\_number\_contig-100\_962.98882SRS077086\_contig\_number\_13341SRS077127\_contig\_number\_2734SRS077194\_contig\_number\_85SRS077194\_contig\_number\_912SRS077194\_contig\_number\_10545SRS077231\_contig\_number\_3873SRS077294\_contig\_number\_19572SRS077335\_contig\_number\_3949SRS077454\_contig\_number\_3518SRS077502\_contig\_number\_6987SRS077552\_contig\_number\_15003SRS077641\_contig\_number\_23721SRS077730\_contig\_number\_17654SRS077849\_contig\_number\_32244SRS078176\_contig\_number\_23154SRS097958\_contig\_number\_274SRS098061\_contig\_number\_8307SRS098073\_contig\_number\_18539SRS098655\_contig\_number\_contig-100\_988.90205SRS098717\_contig\_number\_5610SRS098881\_contig\_number\_27309SRS100021\_contig\_number\_11865SRS101376\_contig\_number\_25859SRS101433\_contig\_number\_2275SRS103987\_contig\_number\_32274SRS104036\_contig\_number\_6500SRS1041036\_contig\_number\_2690SRS1041037\_contig\_number\_16280SRS1041038\_contig\_number\_12707SRS1041039\_contig\_number\_29766SRS1041090\_contig\_number\_8017SRS1041092\_contig\_number\_8001SRS1041118\_contig\_number\_15996SRS1041129\_contig\_number\_16470SRS1041130\_contig\_number\_contig-100\_642.50582SRS1041133\_contig\_number\_2294SRS1041134\_contig\_number\_11126SRS1041136\_contig\_number\_9275SRS1041137\_contig\_number\_33828SRS1041141\_contig\_number\_15300SRS1041142\_contig\_number\_778SRS1041145\_contig\_number\_40845SRS1041146\_contig\_number\_1054SRS1041157\_contig\_number\_7364SRS104311\_contig\_number\_65SRS104400\_contig\_number\_49275SRS104485\_contig\_number\_1521SRS104636\_contig\_number\_25344SRS104693\_contig\_number\_12446SRS104912\_contig\_number\_16285SRS104975\_contig\_number\_25440SRS105082\_contig\_number\_18478SRS105153\_contig\_number\_19444SRS1054716\_contig\_number\_12167SRS1054928\_contig\_number\_contig-100\_3645.32479SRS1055034\_contig\_number\_contig-100\_1233.83960SRS1055043\_contig\_number\_contig-100\_1325.159436SRS1055049\_contig\_number\_17394SRS1055056\_contig\_number\_13505SRS1055069\_contig\_number\_16574SRS1055099\_contig\_number\_14416SRS140492\_contig\_number\_29178SRS140513\_contig\_number\_15673SRS142503\_contig\_number\_1320SRS142542\_contig\_number\_26077SRS142599\_contig\_number\_7892SRS142781\_contig\_number\_10159SRS142890\_contig\_number\_39201SRS142923\_contig\_number\_22945SRS142980\_contig\_number\_contig-100\_624.52627SRS143070\_contig\_number\_17946SRS143148\_contig\_number\_5646SRS143181\_contig\_number\_14602SRS143342\_contig\_number\_7453SRS143417\_contig\_number\_10225SRS143466\_contig\_number\_20733SRS143523\_contig\_number\_25198SRS143722\_contig\_number\_15518SRS143876\_contig\_number\_33860SRS143895\_contig\_number\_179SRS143991\_contig\_number\_28013SRS144135\_contig\_number\_2684SRS144183\_contig\_number\_34399SRS144297\_contig\_number\_13970SRS144506\_contig\_number\_51649SRS144537\_contig\_number\_18051SRS144603\_contig\_number\_17793SRS144714\_contig\_number\_1216SRS144753\_contig\_number\_12321SRS145497\_contig\_number\_20764SRS146764\_contig\_number\_32960SRS146812\_contig\_number\_22927SRS146813\_contig\_number\_8825SRS146888\_contig\_number\_10126SRS147022\_contig\_number\_10277SRS147039\_contig\_number\_5008SRS147139\_contig\_number\_26112SRS147271\_contig\_number\_41617SRS147346\_contig\_number\_47647SRS147377\_contig\_number\_10687SRS147425\_contig\_number\_contig-100\_422.82452SRS147557\_contig\_number\_34693SRS147614\_contig\_number\_10131SRS147653\_contig\_number\_6527SRS147766\_contig\_number\_19886SRS147919\_contig\_number\_1464SRS147977\_contig\_number\_9950SRS148091\_contig\_number\_14826SRS148159\_contig\_number\_12589SRS148196\_contig\_number\_30006SRS148253\_contig\_number\_8754SRS148319\_contig\_number\_17659SRS148424\_contig\_number\_contig-100\_1955.187577SRS148817\_contig\_number\_3673SRS148874\_contig\_number\_12816SRS149075\_contig\_number\_11493SRS149181\_contig\_number\_1604SRS149244\_contig\_number\_12302SRS149325\_contig\_number\_16481SRS149784\_contig\_number\_7851SRS149879\_contig\_number\_19359SRS150029\_contig\_number\_2679SRS892882\_contig\_number\_17076SRS893170\_contig\_number\_4037SRS893172\_contig\_number\_contig-100\_1341.85449SRS893187\_contig\_number\_9799SRS893230\_contig\_number\_12728SRS893231\_contig\_number\_5390SRS893270\_contig\_number\_10667SRS893279\_contig\_number\_16795SRS893300\_contig\_number\_11648SRS893327\_contig\_number\_12044SRS893358\_contig\_number\_14793SRS893366\_contig\_number\_contig-100\_1448.156899SRS893373\_contig\_number\_13862SRS893378\_contig\_number\_7097SRS893383\_contig\_number\_37178SRS971275\_contig\_number\_36327SRS971276\_contig\_number\_36210
853
species
331
